# Comparison of Vaginal Dinoprostone Pessary With Transcervical Balloon Catheter Plus Vaginal Misoprostol for Pre-induction Cervical Ripening: A Randomized Trial

**DOI:** 10.7759/cureus.42261

**Published:** 2023-07-21

**Authors:** Kanagavarshani M, Vanita Jain, Aashima Arora, Jogender Kumar

**Affiliations:** 1 Obstetrics and Gynaecology, Postgraduate Institute of Medical Education and Research, Chandigarh, IND; 2 Pediatric Medicine, Postgraduate Institute of Medical Education and Research, Chandigarh, IND

**Keywords:** labor, rupture of membranes, augmentation of labor, cervical ripening, induction of labor, foley, foley plus misoprostol, dinoprostone pessary

## Abstract

Aims and Objectives

To compare the safety and efficacy of dinoprostone pessary with Foley plus vaginal misoprostol for cervical ripening.

Materials and Methods

We randomized 115 women to the pessary or Foley plus misoprostol group. Pessary was inserted for 24 hours, and in the Foley plus misoprostol group, intravaginal misoprostol 25 mcg was administered along with trans-cervical Foley insertion and repeated every six hours to a maximum dose of 100 mcg. Singleton pregnancies requiring labor induction at more than 34 weeks with a Bishop score of <6 were included. Study outcomes included induction-delivery interval (IDI), mode of delivery, change in the Bishop score, need for oxytocin augmentation, and patient discomfort as assessed by visual analog score.

Results

The IDI was similar between the groups (pessary vs Foley plus misoprostol; 21.27 vs 21.10 hours, p = 0.9). The mean change in the Bishop score and need for augmentation with oxytocin was significantly more in the Foley plus misoprostol group compared to pessary (2.72 vs 1.94, p = 0.001; 89.7% vs 57.9%, p = 0.0001). Pessary was better tolerated compared to Foley plus misoprostol (VAS 7.8 vs 6.68, p = 0.0001). Mode of delivery and maternal and neonatal outcomes showed no difference.

Conclusion

There was no significant difference between pessary and Foley plus misoprostol in the IDI and mode of delivery. Pessary was better tolerated, and augmentation with oxytocin was required less often. Foley plus misoprostol caused a faster change in the Bishop score, but oxytocin augmentation was used more often. Maternal and neonatal outcomes were similar.

## Introduction

Induction of labor (IOL) is a routine procedure in modern-day obstetrics. IOL is done when the risks of continuing pregnancy outweigh the benefits to the mother or the fetus [1]. The rate of IOL in the Postgraduate Institute of Medical Education and Research (PGIMER), Chandigarh, India, a tertiary care hospital in North India, was 27.1% in 2021, with the most common indications being pre-eclampsia, pre-labor rupture of membranes, and cholestasis of pregnancy. The predictors of a successful induction are multi-parity, tall stature, low body mass index (BMI), a fetus with low birth weight, and increased gestational age [2]. One of the important predictors is cervical status as graded by the Bishop score, with the most important parameter being dilatation [3]. IOL done with oxytocin alone in an unripe cervix without pre-induction cervical ripening decreases the successful vaginal delivery rate [4]. Hence, pre-induction cervical ripening is important.

Cervical ripening is a slow process that happens throughout pregnancy and is hastened just before labor. It is a process that is mediated by cytokines and interleukins like IL-8, IL-6, and G-CSF. Hormones like estrogen and prostaglandins are also known to play a role. A study conducted by Sennström et al. clearly shows that cervical ripening is an inflammatory process [5]. As the cervix ripens, the solubility of collagen increases, and proteoglycans are broken down. Inflammatory mediators like interleukins stimulate the activity of neutrophils. Neutrophils release enzymes like collagenase and elastase, which degrade collagen and proteoglycans [5].

There are various methods for cervical ripening, namely mechanical, pharmacological, surgical, or a combination of these. Dinoprostone vaginal pessary is a controlled-release drug device containing 10 mg of dinoprostone resulting in a continuous release of dinoprostone at the rate of 0.3 mg/hour throughout 24 hours. The ease of administration and need for fewer vaginal examinations make it comfortable for the patients. A study comparing intracervical gel vs vaginal pessary showed that pessary had a higher mean change in the Bishop score than intracervical gel (3.2 ± 3.1 vs 1.8 ± 1.9, p = 0.01) [6]. The mean time to delivery was not significantly different between pessary and gel (28.3 vs 24 hours, p = 0.19), and also, the percentage of patients requiring cesarean delivery was similar. 

In another study conducted by Triglia et al., vaginal pessary was compared with gel in patients with a very unfavorable cervix Bishop score of <4 [7]. The rate of vaginal delivery was higher in the pessary group compared to gel (72% vs 54%) with a significant difference (p = 0.03). No statistically significant difference was found in the rate of cesarean section (25% vs 31%). In either group, none of the participants had hyperstimulation, and no significant maternal or neonatal side effects were noted. Though the cost was higher for a single dose of a pessary (pessary, 79.02€; gel, 64.61€), the use of pessary had a lower median cost of pharmaceutical preparations per woman. Thus, this study showed that pessary was more successful in induction in women with an unfavorable cervix.

Foley catheter plus misoprostol is a combination of a mechanical and a pharmacological method. Foley dilates the cervix mechanically, and misoprostol softens the cervix. Both act synergistically to improve different parameters, i.e., Foley acts on dilatation and misoprostol on effacement; hence, they may be more effective when used together [8]. Trials comparing Foley plus vaginal misoprostol vs misoprostol alone have shown that induction-delivery interval (IDI) was reduced in the combination group compared with the only misoprostol group. However, no studies were done comparing dinoprostone pessary with Foley plus vaginal misoprostol. Given the scarce research and evidence available comparing these two methods, we chose to compare them for cervical ripening.

The aim of our study was to compare the efficacy and safety of intravaginal dinoprostone pessary with Foley plus vaginal misoprostol for pre-induction cervical ripening. The primary objective was to compare the IDI. Secondary objectives were to compare the change in the Bishop score, mode of delivery, rate of uterine hyperstimulation, neonatal outcome, maternal endometritis, and discomfort during the procedure using a visual analog scale (VAS).

## Materials and methods

This prospective randomized trial was undertaken in the Department of Obstetrics and Gynaecology and the Department of Neonatology of PGIMER, Chandigarh, India. The trial was registered on the CTRI website under trial number CTRI/2021/09/036894 and cleared by the ethical committee of our institute with reference number NK/7666/MD/514. A total of 115 women admitted for IOL were recruited. Inclusion criteria were cephalic presentation, gestational age >34 weeks, a Bishop score of <6, reactive nonstress test, absence of uterine contractions, and intact membranes. Subjects with the presence of uterine scar, antepartum hemorrhage, intrauterine fetal demise, severe fetal growth restriction (estimated fetal birth weight <3rd percentile), severe pre-eclampsia, parity >3, maternal medical disorders, uncontrolled diabetes mellitus, and history of uterine hyperstimulation and glaucoma were excluded.

Written informed consent was obtained, and women were randomized into two groups based on a computer-generated randomization table. A detailed history was taken and recorded on pre-designed proforma. A general physical, an abdominal, and a vaginal examination were carried out, and findings were noted. Women underwent cervical ripening either with dinoprostone pessary or Foley catheter and vaginal misoprostol.

Dinoprostone pessary group

Taking aseptic precautions, the pessary was inserted digitally into the posterior vaginal fornix. The women were told to lie recumbent for 30 minutes after insertion. Following this, they were asked to grade the discomfort experienced during the procedure on a VAS.

Foley plus misoprostol group

A 16 G Foley catheter was inserted into the cervix, and the balloon was filled with 30 mL of saline. The catheter was strapped to the woman’s thigh. Simultaneously, 25 mcg of misoprostol was placed in the posterior fornix. Following this, women were asked to grade the discomfort experienced during the procedure on a VAS.

Monitoring

Cervical assessment for the Bishop score was done every six hours. The misoprostol dose was repeated every six hours till a maximum of 100 mcg or till the women achieved uterine contractions, and the Bishop score was assessed to be >6.

Removal

The inducing agents were removed when the Bishop score was >6 and any signs of hyperstimulation, fetal heart rate (FHR) abnormalities, rupture of membrane, or any maternal systemic side effects occurred. When the Foley catheter was expelled spontaneously, cervical reassessment was performed. No attempt was made to reinsert the catheter. Both the pessary and catheter were removed after 24 hours when not expelled.

Labor monitoring

When the Bishop score was >6 and the woman had adequate uterine contractions, oxytocin was not started. When contractions were inadequate (less than three contractions lasting for 45 seconds in 10 minutes), oxytocin infusion was started at the rate of 3 mIU/hour and increased by 3 mIU/hour every 30 minutes to a maximum of 42 mIU/hour. The cervical assessment was done every four to six hours or when the attending obstetrician deemed it necessary. Artificial rupture of membranes (ARM) was done when there were inadequate uterine contractions despite augmentation with oxytocin at a maximum infusion rate or when FHR was non-reassuring. In low-risk pregnancies, fetal heart sound was auscultated every 30 minutes in the first stage and every five minutes in the second stage. When there were any abnormalities in FHR, a cardiotocograph (CTG) trace was taken. In high-risk pregnancies and in those requiring augmentation with oxytocin, continuous CTG monitoring was done. When the woman did not deliver after 24 hours, the doctor in charge decided further management. Primary and secondary objectives were noted.

The clinical details of all the participants included in the study were coded and entered in the Microsoft Excel sheet. The statistical analysis was performed by IBM SPSS Statistics, version 23.0 (IBM Corp., Armonk, NY) software. The continuous variables were recorded and analyzed in the form of mean + standard deviation and median + interquartile range. The categorical variables were analyzed in terms of frequencies and percentages. The independent sample t-test was used to compare groups having data with continuous distribution. The non-parametric tests, like the Wilcoxon-Mann-Whitney U test, were used for data that did not have a normal distribution. The comparison of groups with categorical data was done using the chi-square test. The level of statistical significance was kept at p < 0.05.

## Results

**Figure 1:**
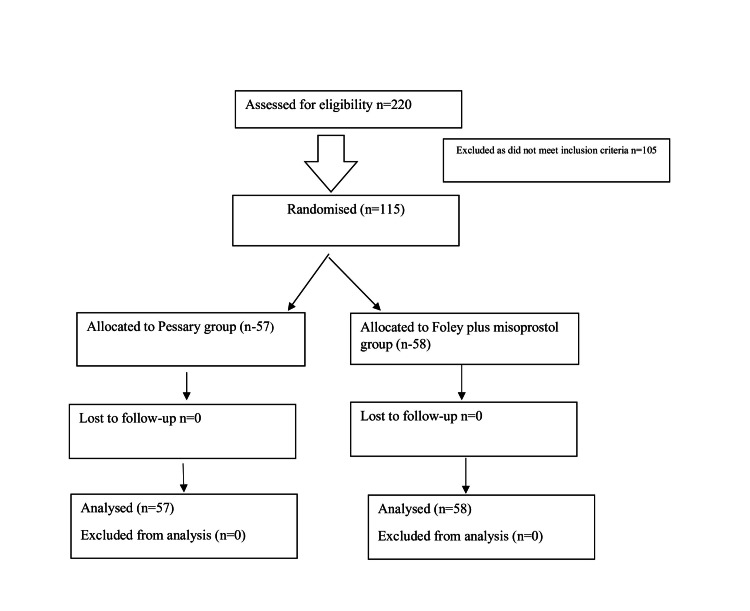
A CONSORT flow diagram to show the inclusion of patients

Between July 2021 and July 2022, 115 pregnant women were recruited in our study and were randomized to the pessary or Foley plus misoprostol group. A CONSORT diagram shows the inclusion of the patient (Figure 1).

Both groups were comparable in terms of baseline characteristics like mean age, parity, and BMI, as depicted in Table 1. The most common indication for induction was intrahepatic cholestasis of pregnancy in both groups.

**Table 1:** Baseline parameters of the two groups IOL, induction of labor; FGR, fetal growth restriction; HDP, hypertensive disorders of pregnancy; ICP, intra-hepatic cholestasis of pregnancy; GDM, gestational diabetes mellitus; EFW, estimated fetal weight

Parameters	Pessary group (n = 57), N (%) or mean + SD	Foley plus misoprostol group (n = 58), N (%) or mean + SD	p-value
Age (years)	27.71 ± 3.92	28.65 ± 4.97	0.2
Period of gestation (weeks)	37.88 ± 1.21	37.61 ± 0.91	0.1
Body mass index (kg/m^2^)	24.49 ± 3.05	24.98 ± 3.23	0.3
Primipara	39 (68.4%)	36 (62.1%)	0.475
Multipara	18 (31.6%)	22 (37.9%)	
Indication for IOL-GDM	6 (10.5%)	3 (5.2%)	0.2
Indication for IOL-HDP	16 (28.1%)	17 (29.3%)	0.8
Indication for IOL-ICP	21 (36.8%)	35 (60.3%)	0.012
Indication for IOL-post-datism	6 (10.5%)	23 (39.7%)	0.4
Indication for IOL-oligohydramnios	4 (7%)	3 (5.2%)	0.6
Indication for IOL-decreased fetal movements	2 (3.5%)	0	0.1
Indication for IOL-FGR (EFW, 3rd-10th percentile)	7 (12.3%)	4 (6.9%)	0.3

The difference in IDI was not statistically significant between the pessary and Foley plus misoprostol group (21.27 + 11.23 vs 21.10 + 11.13 hours, p = 0.935), as shown in Table 2.

**Table 2:** Primary and secondary outcomes between the groups PV, per vaginal; VAS, visual analog scale; IDI, induction-delivery interval

	Pessary group, N (%) or mean ± SD	Foley plus misoprostol group, mean ± SD	p-value
Primary outcome IDI (hours)	21.27 ± 11.23	21.10 ± 11.13	0.9
Change in Bishop score after six hours (between first and second PV examination)	1.94 ± 2.33	2.72 ± 1.65	0.001
Time taken to change from an unfavorable to a favorable Bishop score (hours)	12.56 ± 7.36	7.36 ± 3.88	0.0001
VAS	6.68 ± 1.15	7.86 ± 0.78	0.0001
Rate of hyperstimulation	3 (5.3%)	0	0.075

The Bishop score on baseline examination was comparable in both groups (2.84 ± 0.84 vs 2.91 ± 1.14, p = 0.297). Women in the Foley plus misoprostol group had a higher mean change in the Bishop score after six hours compared to the pessary group (2.72 ± 1.65 vs 1.94 ± 2.33, p = 0.001). The time taken to change from an unfavorable to a favorable Bishop score was also significantly less in the Foley plus misoprostol group (7.36 ± 3.88 vs 12.56 ± 7.36 hours, p = 0.0001). In the Foley plus misoprostol group, women showed higher discomfort as compared to the pessary group (7.86 ± 0.78 vs 6.68 ± 1.15, p = 0.0001). Three women in the pessary group developed hyperstimulation; however, it was not statistically significant.

The delivery details and the comparison between the groups are given in Table 3.

**Table 3:** Details of delivery and comparison between the groups AOL, augmentation of labor; ARM, artificial rupture of membranes

	Pessary group (n = 57), N (%) or mean ± SD	Foley plus misoprostol group (n = 58), N (%) or mean ± SD	p-value
Need for ARM	21 (37.5%)	35 (60.3%)	0.015
Duration of stages 1 and 2 (hours)	9.74 ± 3.95	10.59 ± 5.56	0.4
Duration of stage 3 (hours)	2.51 ± 0.84	2.49 ± 0.66	0.8
Need for AOL	33 (57.9%)	52 (89.7%)	0.0001
Duration of use of interventional agent	13.23 ± 6.62	6.92 ± 3.49	0.0001
Vaginal delivery	47 (82.5%)	43 (74.1%)	0.1
Instrumental delivery	0	3 (5.2%)	
Cesarean delivery	10 (17.5%)	12 (20.7%)	

The mean duration of pessary use was comparatively longer compared to Foley plus misoprostol (13.23 vs 6.92 hours, p = 0.0001). Seven women in the pessary group required removal of pessary after 24 hours of use, whereas there were none in the Foley plus misoprostol group (23% vs 0%, p = 0.005). The number of doses of misoprostol required in the combination group was also assessed. In all, 45% of women required only a single dose of misoprostol, while 10% required two doses and 3% required three doses. None of the women needed four doses. The need for augmentation, defined as inadequate uterine contractions with a Bishop score of >6, was more in the Foley plus misoprostol group compared to the pessary group (89.7% vs 57.9%, p = 0.001). The duration of the first and second stages of labor was not statistically different between the two groups (9.74 vs 10.59 hours, p = 0.400). The rate of ARM was more in the Foley plus misoprostol group compared to the pessary group (60.3% vs 37.5%, p = 0.015). The mode of delivery was not significantly different between both groups.

The neonatal outcome was compared in terms of the Apgar score, admission to the neonatal intensive care unit (NICU), neonatal jaundice, transient tachypnoea of newborn, need for resuscitation, presence of meconium-stained liquor (MSL), and perinatal asphyxia, as shown in Table 4. Neonatal outcome was comparable between the two groups. Two neonates each in the pessary and Foley plus misoprostol group required bag and mask ventilation. Three neonates in the Foley plus misoprostol group had MSL, while there were none in the pessary group; however, the difference was not statistically significant (p = 0.082).

**Table 4:** Comparison of neonatal outcomes between both groups Apgar, appearance, pulse, grimace, activity, and respiration; NICU, neonatal intensive care unit; NNN, neonatal nursery; MSL, meconium-stained liquor; TTN, transient tachypnoea of newborn

	Pessary group (n = 57), N (%) or mean + SD	Foley plus misoprostol group (n = 58), N (%) or mean + SD	p-value
Birthweight (grams)	2739.91 + 479.88	2814.20 + 475.64	0.4
Apgar at 1 minute	8 (7,9)	8 (6,9)	0.3
Apgar at 5 minutes	9 (8,10)	9 (8,10)	0.1
Neonatal jaundice	2 (3.5%)	1 (1.7%)	0.5
Need for resuscitation	2 (3.5%)	2 (3.4%)	0.9
MSL (vigorous)	0	3 (5.2%)	0.08
TTN	1	1	0.9
Perinatal asphyxia	0	0	Not applicable

One woman in the pessary group and three women in the Foley plus misoprostol group had a fever in the postpartum period (p = 0.317), while none of the women had foul-smelling lochia or lower abdominal pain in both groups. Thus, there was no significant difference in maternal postpartum status between groups.

## Discussion

In the present study, we compared dinoprostone pessary with a Foley catheter plus vaginal misoprostol. We recruited 115 women, out of which 57 got randomized to the pessary group and 58 to the Foley plus misoprostol group. The main results of our study were that pessary was more comfortable to women, and the need for augmentation with oxytocin was lesser in the pessary group, whereas Foley plus misoprostol caused faster ripening of the cervix but needed augmentation by oxytocin. Both methods were comparable in terms of IDI, type of delivery, and maternal and neonatal outcomes.

Our study included women with an unfavorable cervix (Bishop score, <6), and the indication for IOL was noted for each patient in both groups. Intrahepatic cholestasis of pregnancy was noted to be the most common indication for IOL. This can be explained by the higher incidence of intra-hepatic cholestasis of pregnancy in the Asian population, which is around 1.24% compared to 0.62% in the Caucasian population [9].

The induction to the delivery interval was comparable in both groups. The Foley plus misoprostol group had a higher mean change in the Bishop score after six hours compared to the pessary group. The time to change from an unfavorable to a favorable bishop score was also significantly less in the Foley plus misoprostol group. This is comparable to a study conducted by Carbone et al. [10], where they found that the mean duration of induction to complete dilatation is shorter in the combination group compared to the vaginal misoprostol alone group (13.7 vs 17.1 hours, p = 0.03). Thus, Foley plus misoprostol, being a combination method, had a faster action on cervical ripening.

We measured the discomfort by a VAS, where women did a subjective ranking from 0 to 10 about the discomfort they faced. VAS was not assessed in any of the previously published studies to the best of the author’s knowledge. Women in the Foley plus misoprostol group showed higher discomfort compared to the pessary group. This could probably be because the pessary is inserted digitally, while a vaginal speculum is required for the insertion of Foley, and saline is instilled to inflate the Foley bulb, which causes mechanical stretching of the cervix causing pain.

Three women in the pessary group developed hyperstimulation; two underwent cesarean delivery, while one had a vaginal delivery. This observation implies that the main action of pessary could be uterine stimulation rather than direct action on cervical ripening. In a non-inferiority randomized control trial conducted by Gaudineau et al., where they compared the efficacy of pessary vs vaginal misoprostol 25 mcg inserted every four hours for pre-induction cervical ripening, no significant difference was noted between the groups in terms of uterine hyperstimulation (3.2 vs 3, p = 0.785) [11]. In a study conducted by Kho et al. [12], comparing dinoprostone vaginal pessary vs dinoprostone gel, uterine hyperstimulation was comparatively higher in the pessary group (4.5% vs 2.2%, RR = 1.9), although it was not statically significant. However, more women in the pessary group required tocolysis or cesarean delivery for hyperstimulation compared to the gel group (1.8% vs 0.2%, RR = 8.4; 1.4 vs 0.2%, RR = 6.5). The rate of hyperstimulation in the Foley plus misoprostol group, when compared with misoprostol in a study by Lanka et al., showed that it was significantly less in the combination group compared to vaginal misoprostol alone (7.94% vs 39.68%, p ≤ 0.001) [13]. This is consistent with our study and may be due to the lesser repeat dose of misoprostol required in the combination group. Thus, one of the advantages of Foley plus misoprostol is the reduced side effects of misoprostol.

The mean duration of pessary was comparatively longer compared to Foley plus misoprostol, which again shows that Foley plus misoprostol had faster action. The need for augmentation of labor was higher in the Foley plus misoprostol group compared to the pessary group (89.7% vs 57.9%, p = 0.0001). In a study conducted by Lanka et al. [13], the rate of augmentation by oxytocin was 55.56% in the Foley plus misoprostol group, which was lower compared to our study. This could be explained by the fact that in that study, 25 mcg of misoprostol was repeated at an interval of four hours to a maximum of eight doses. In another study by Carbone et al. [10], 25 mcg of misoprostol was repeated every six hours to a maximum of 100 mcg; similar to our study, the need for oxytocin augmentation was 82.1%. Thus, the dose and frequency of repeating misoprostol seem to affect the requirement of oxytocin. In the pessary group, the requirement of augmentation by oxytocin was significantly less, which was comparable to a study conducted by Senthilvel and Vijayakrishnan [14], in which the need for oxytocin was significantly less in the pessary group compared to gel (37% vs 51%, p = 0.029). This shows that pessary slowly releases a constant amount of prostaglandins simulating natural labor.

The duration of the first and second stages of labor was not statistically different between the two groups. More women in the Foley plus misoprostol group required amniotomy for labor augmentation compared to pessary (60.3% vs 37.5%, p = 0.015). This is comparable to a study conducted by Sulaiman et al. [15], where the need for amniotomy was 91% in the Foley group compared to only 63% in the pessary group (p = 0.004). This may be due to cervical ripening by Foley without inducing any uterine contractions, whereas pessary causes both ripening and uterine contractions simultaneously. There was no difference in the mode of delivery between the groups. Neonatal outcome and maternal endometritis rate were comparable between the two groups.

The strengths of our study were that this is the first study comparing pessary with Foley plus misoprostol to the best of the author’s knowledge. We followed a strict protocol with a 24-hour limit for both groups, and no secondary method of ripening was used, patient comfort was assessed, and the maternal endometritis rate was also compared.

Limitations were that it was an open-labeled trial; blinding could not be done, as the ripening agents are grossly different and have different methods of insertion. The provider could not be blinded to the method or the procedure. Lastly, the sample size was not large enough to compare the safety and effectiveness of the induction agents.

## Conclusions

There was no significant difference between pessary and Foley plus misoprostol in the IDI and mode of delivery. The pessary was better tolerated and resulted in spontaneous labor. Foley plus misoprostol caused a faster change in the Bishop score but required augmentation by oxytocin. Both groups were comparable in terms of maternal and neonatal outcomes.
